# Challenging encounters as experienced by registered nurses new to the emergency medical service: explored by using the theory of communities of practice

**DOI:** 10.1007/s10459-018-9862-x

**Published:** 2018-11-15

**Authors:** Anna Hörberg, Veronica Lindström, Max Scheja, Helen Conte, Susanne Kalén

**Affiliations:** 1 Department of Clinical Sciences and Education, Södersjukhuset, Karolinska Institutet, Sjukhusbacken 10, 118 83 Stockholm, Sweden; 2 Academic EMS Stockholm, Stockholm, Sweden; 3 Department of Neurobiology Care Sciences and Society, Division of Nursing, Karolinska Institutet, Stockholm, Sweden; 4 Department of Education, Stockholm University, Stockholm, Sweden; 5 Stockholm City Council, Stockholm, Sweden

**Keywords:** Ambulances, Communities of practice, Emergency medical service (EMS), Mentoring, Nurses, Professional role, Staff development

## Abstract

The aim of this study was to explore challenging encounters experienced by registered nurses (RN) during their first year in the emergency medical service by using the social learning theory of communities of practice. During the first year in a new professional practice, a new RN experiences a transition during which the new professional identity is being formed. This is a challenging and demanding period of time. According to the learning theory of communities of practice by Lave and Wenger, individuals’ learning and development in a new professional practice occurs through participation in social activity and is influenced by context. This study is based on the qualitative data from semi-structured interviews. Thirty-two RNs working in the Swedish emergency medical service were interviewed via telephone during the spring of 2017. A qualitative content analysis with deductive reasoning of the interviews was used. The analysis process generated the main category; *New RNs participation is challenged by unpredictability and uncertainty in practice*. The main category was based on three generic categories; *Loneliness in an unpredictable context, Uncertainty about the team,* and *Uncertainty in action*. The challenges new RNs encounter during the first year relate to all three dimensions of a community of practice; mutual engagement, joint enterprise and shared repertoire. The encountered challenges also relate to the EMS context. Taking into account all these aspects when designing support models for RN’s professional development may be advantageous for creating positive development for RNs new to the EMS and/or similar practices.

## Introduction

Registered nurses (RN) worldwide report difficulties in adapting to unfamiliar circumstances when new to a professional practice, despite thorough, formal education and professionally tailored preparation (Gerrish 2000).

The reality of professional work and the RNs’ expectations do not always harmonize, negatively affecting quality of care, patient outcome and turnover rates (Kramer 1974; Kramer et al. 2013). The emergency medical service (EMS) is no exception (Patterson et al. 2010). In the EMS, a new professional need to adapt to work with limited resources in highly varied settings where space may be constrained, light might be limited and footing unstable. In addition, EMS professionals work with critically ill patients in sub-optimal positions and with unpredictable bystanders (Carter and Thompson 2015; Mausz and Tavares 2017). To our knowledge there is a lack of knowledge about how new professionals develop and adapt to the challenges of a professional practice like the EMS. The aim of this study was to explore challenging encounters experienced by RNs during their first year in the EMS using the social learning theory of communities of practice.

### Background

During the first year in a new professional practice a RN goes through a transition where the new professional identity is developed (Brown and Olshansky 1997; Hörberg et al. 2017b). Although difficult to overcome, the challenges of transition are essential for professional development and the acquisition of new knowledge and skills (Barnes 2015; Farnell and Dawson 2006; Lave and Wenger 1991).

Transition is a concept that involves the way people respond to changes in their life world (Meleis 1975). Transition, a dynamic process entailing change and adaption occurring over time, has been described as a journey from one position in life to another, and as a reconstruction of self-identity (Barnes 2015; Kralik 2006; MacLellan et al. 2015). Transition is usually described in stages, the first stage entails the new RN letting go of the old professional identity and may be an emotional period marked by stress, confusion and anxiety (Barnes 2015; Bridges 2009; Brown and Olshansky 1997). The second stage commences when the old professional identity has been abandoned but the new is not yet accepted. This second stage has been described as a psychological no-man’s land, a limbo between two identities. If the responsibilities placed on the new RN during the second stage are too great, self-confidence may be affected (Bridges 2009). The third and last stage begins when the new RN has made significant advances in becoming more confident and feels more legitimate in the new professional practice (Brown and Olshansky 1997). At this stage, a new identity with new values and attitudes and is being formed.

Another challenging aspect involved in the transition into a new professional identity is finding one’s own part to play in the new professional practice and becoming a legitimate member of the team (Bridges 2009; Conte et al. 2015b). Becoming a legitimate member of a team is essential for successful team work, patient safety and retention (Numminen et al. 2015).

Our research stems from the epistemological notion that learning and professional development occur through participation with and in a team. In the EMS, it has been suggested that the team has profound influence on the new professionals’ development (Hörberg et al. 2017b). Therefore, the social learning theory of communities of practice (CoP) (Lave and Wenger 1991) has been used in our study to represent one view of the development of a new professional identity during the first year in a new professional practice.

#### Communities of practice

The theory of CoP is based on the assumption that activity is always situated, and learning in a new professional practice is never simply a matter of the acquisition of knowledge and/or skills (Lave and Wenger 1991). As newcomers strive to become respected members of the new professional community, they interact with new people, learn new skills and routines, and adapt to the new culture. Between the individual, the activity and the practice in which the individual engages, there is a constant interplay that shapes not only what the individual does but also who they are and how they interpret what they do (Wenger 1998). We will refer to this interplay in terms of professional development. In this sense, professional development can be said to involve learning to become a member of a specific professional practice and the transition into a new professional identity.

CoPs are everywhere and a person can be engaged in a number of them, for example as a member of a professional practice and as a family member (Cruess et al. 2017; Wenger 2000). While participation is the fundamental key to development, a person enters a CoP as a legitimate peripheral participant (Lave and Wenger 1991). As a person becomes more engaged in practice they become more knowledgeable and skilful and move from legitimate peripheral participation to full participation. A CoP can be described as the place where people share, develop and negotiate their understanding of the world. There are three dimensions that together constitute a CoP, mutual engagement, joint enterprise, and shared repertoire (Wenger 2000).

#### Mutual engagement

Being in a CoP is not just a matter of having a title or belonging to an organisation. It is the participant’s *mutual engagement* that defines the CoP. This does not necessarily entail homogeneity. What makes mutual engagement possible is as much diversity as it is homogeneity. It involves an individual’s competence but also the competence of others (Wenger 1998). In an EMS team, the two professionals may have different educational backgrounds, however their mutual engagement is to care for the out-of-hospital patients.

#### Joint enterprise

What constitutes a particular CoP is negotiated by its participants into a *joint enterprise*. Defining a joint enterprise is a process that is as generative as it is constraining. Metaphorically speaking, it is like a dance between accepting new knowledge and holding on to old beliefs (Wenger 1998). The negotiation of enterprise involves knowing what matters and what does not, what to talk about and what to leave unsaid, what to display and what to withhold, and recognising when actions are good enough and when they need improvement (Wenger 1998). There is a constant and ongoing process of negotiation between the participants and this gives rise to relations of mutual accountability. In the EMS team this can mean that members mutually trust each other to do what is best for the patient.

#### Shared repertoire

The third dimension of a CoP is *shared repertoire*. Over time, the negotiated pursuit of a joint enterprise creates resources for that same enterprise. These can include routines, language, tools, symbols and genres (Wenger 1998). In the EMS, symbols of the CoP include the shared uniform, the guidelines provided, medical equipment and ways of talking e.g. a dark humour (Rosenberg 1991).

#### The context of the emergency medical services

Around the world, EMS professionals seem to be facing increasingly more demanding workloads and professionals are leaving the profession in increasingly higher rates (Cooper and Grant 2009; Evans et al. 2013; O’Hara et al. 2015; Patterson et al. 2010; Suserud 2005; Tohira et al. 2014). The reality of a professional practice like the EMS is far from ideal. Professionals in EMSs around the world work in teams of two with limited resources and meet critically ill or injured patients in highly varying settings. It has been described as a practice where the only thing that professionals can prepare for is to be unprepared (Sundström and Dahlberg 2012). Without understanding the challenges that RNs new to the EMS encounter, providing support within the profession will be difficult.

In Sweden, where this study was conducted, the national regulation dictates that an ambulance in the EMS needs to be staffed by at least one RN (SOSFS 2009). Registered nurses in Sweden hold a baccalaureate degree. While not a national requirement, some regions in Sweden only employ RNs holding a specialist degree in nursing, preferably in prehospital emergency care (Lindström et al. 2015). A specialist degree in nursing is a protected occupational title achieved by an additional year of post graduate education. While the educational content of this additional year emphasises medical knowledge, focus is also centred on nursing knowledge as well as contextual knowledge (Sjolin et al. 2014). In some universities in Sweden, the specialist nurse education program also leads to a master’s degree in nursing science (ibid). Other professions represented in the EMS are emergency medical technicians (EMT) and physicians. There is no national regulation of the presence of these professions. Physicians, for example, may be available only via telephone or in special units such as a helicopter (Lindström et al. 2015). When an EMT constitutes one member of the ambulance team, EMTs are always paired with an RN, where the RN has the responsibility for the care (Wihlborg et al. 2014).

By exploring challenging encounters by using the social learning theory of communities of practice, this research contributes to the field of knowledge about new professionals’ development in a professional practice like the EMS and other ambulatory care practices.

### Design

This is a qualitative study where the theory of CoP (Lave and Wenger 1991) have been used as analytical frame. Analysis was based on the qualitative content analysis process described by Elo and Kyngas (2008).

### Informants

Inclusion criteria were RNs or RNs with a specialist degree, working fulltime as the one in the EMS team with the highest level of formal education, and that had been employed within an EMS organisation between 12 and 36 months.

A purposeful sample and snowball technique was used to identify informants (Patton 2002). Region managers and human resource personnel were informed about the study and asked to identify possible informants. The names of the eligible informants that had given consent to be included in the study were e-mailed to the first author (AH). After each interview the interviewed informant was asked if he/she could recommend other possible informants. As a snowball gets bigger with each turn in the snow, the number of eligible participants increased with every interview (Patton 2002). Thirty-two informants agreed to participate (Table 1) representing both RNs and RNs with a specialist degree. All informants had the highest level of formal education in their team. Henceforth, when using the term ‘RN’, we refer to both RNs and RNs with a specialist degree.

**Table 1:** Demographic information of participants adapted from Hörberg et al. (2017a)https://creativecommons.org/licenses/by/4.0/

Demographics		Number (percent)
Total of included participants		32 (100)
Gender	Male	12 (37.5)
	Female	20 (62.5)
Academic degree	Registered nurse	9 (28)
	Specialist nurse (one year additional education)	23 (72)
Geographic region	Urban	16 (50)
	Sub-urban	10 (31)
	Rural	6 (19)
Months of experience (*in the EMS*)	12–24	25 (78)
	> 25	7 (22)
Years of RN experience	< 5	11 (34)
	5–10	15 (47)

### Data collection

Data were collected by telephone interviews during the spring of 2016. The time for the interview was chosen by the informant, and interviews lasted between 6 and 30 min, with a mean time of 14 min. To facilitate dependability, all interviews were performed by the first author, using a semi-structured interview guide. The semi-structured interview guide was developed by all authors, and piloted in a group of seven people, two researchers, three experienced RNs and two novice RNs. The interview guide consisted of three main questions:

1. Can you tell me about a situation during your first year in which you did not experience that you could manage the way you would have liked to?
2. What support would you have desired to manage that particular situation?
3. During your first year, what other support, apart from that you just described, would you have desired?

In this study, data generated from question one, the informants’ stories about experienced challenging encounters, was analyzed. The data generated by the answers from question two and three was analyzed in a study by Hörberg et al. (2017a) that identifies desirable support during the first year in the EMS.

### Ethical considerations

All RNs were informed about the study in written and oral form, participation was voluntary, and all gave consent for the material to be used in this study. Confidentiality was guaranteed and the RNs were informed that they could retract their participation at any time without consequences.

This study was approved by the Regional Ethical Board (Diary number 2015/87-31/5).

### Data analysis

**Fig. 1:**
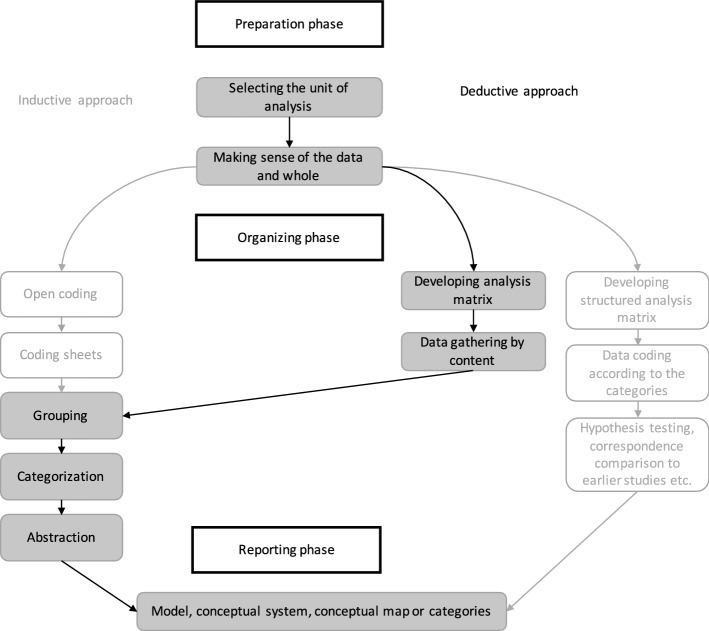
Data analysis process show by the marked trail, adapted from Elo and Kyngäs (2008). Reproduced with permission by Wiley and Sons

The data were analysed by qualitative content analysis using the approach described by Elo and Kyngas (2008) and shown by the marked trail in Fig. 1.

The analysis process was structured by the three phases of preparation, organising and reporting as suggested by Elo and Kyngas (2008).

#### Preparation phase

All interviews were recorded and transcribed verbatim by the first author (AH). The transcribed material was checked for coherence by AH and VL. The transcribed data from question one of the interview guide constituted the unit of analysis. To make sense of the data and of the whole, the interviews were read repeatedly.

#### Organising phase

To identify challenges regarding professional development within the CoP a categorisation matrix based on the three dimensions of a CoP was used (Elo and Kyngas 2008; Wenger 1998) (Table 2). Data was gathered by the described challenges being highlighted, openly coded and grouped accordingly to correspondence with the challenge of mutual engagement, the challenge of a joint enterprise and the challenge of a shared repertoire. Statements that did not fit in any of the challenges of a CoP were sorted as a fourth area labelled as ‘other challenges’. Data analysis was performed by all authors. Four of the authors (AH, HC, MS, SK) were well versed in the theory of CoP while two authors (AH, VL) were knowledgeable about the EMS context. The mix of author knowledge provided both insider and outsider perspectives during the analysis process. Recurrent discussions regarding which content each statement would belong to and categorization were performed until consensus was reached.

**Table 2:** Categorization matrix and example of the analysis process

Content area from theory	Data from interviews	Codes from interviews	Sub-categories developed from codes
The challenge of mutual engagement	…and she (EMT) continued to have tins aggressive attitude and question why they had called for an ambulance at all, meanwhile tins man is taking his last breaths […] I just wanted to sink through the face of the earth, it was so embarrassing. (Informant 2)	Feel embarrassed by the colleague’s attitude	Team conflicts
	I have never gotten any real training in how to assess a neck injury so I asked my colleague for guidance and lie only says, you’re the nurse, it’s your call. (Informant 12)	Unsupportive colleague	Lack of unity
	I was supposed to be MA (medical officer) but I was too new…I couldn’t take any…I couldn’t stop my colleague and say we need to do this and this… (Informant 32)	Not being able to take the leader role	Unclear roles
The challenge of negotiating a joint enterprise	Too me it’s really important, but she didn’t get that, she just kept on having that harsh attitude meanwhile this patient is actually dying in front of us… (Informant 2)	Not shared values with the colleague	View on assignment
	I didn’t agree but he (EMT) has worked so many years so I trusted him […] but when we came to the hospital, they questioned my assessment. (Informant 11)	Not being able to trust the colleague	Team accountability
	I just stood there and took the scolding, that doctor really run me over and I didn’t have enough knowledge to stand up for myself in that situation. (Informant 28)	Not standing up one self	View on own contribution
The challenge of a shared repertoire	I kept looking for that thing to attach it (the CPAP) with…but I couldn’t find it… […] later I understood that I thought it (the CPAP) was supposed to look different than what it did in reality… (Informant 18)	Not knowing how to use the equipment	Lack of knowledge
	I wanted to go to the hospital with the patient but in this case, we had the other patient as well… (Informant 31)	Not being able to do what feels right	Lack of experience
	I drove to an address I thought was the right but when we arrived I realized it was wrong […] I panicked. (Informant 8)	Driving to the wrong address	Making a mistake
Other challenges	It was a traffic accident…eh…on a smaller road […] we knew we were the only available ambulance because everyone else was busy… (Informant 9)	Being the only ambulance on scene	Lack of resources
	It was my first cardiac arrest and I didn’t know all the routines and there was no one I would turn to and ask either. (Informant 7)	No one to ask	Working independently
	There were three adolescents screaming and a lot of people around and…well…I was not prepared for that… (Informant 19)	Not feeling prepared for what to encounter	An unpredictable context

Codes within all of the four areas were grouped and labelled with a descriptive content-characteristic sentence forming twelve sub-categories (Table 2). Of the twelve sub-categories three generic categories were created by grouping sub-categories that were considered as belonging to the same topic under higher order headings. The three generic categories were described by a main category.

#### Reporting phase

**Fig. 2:**
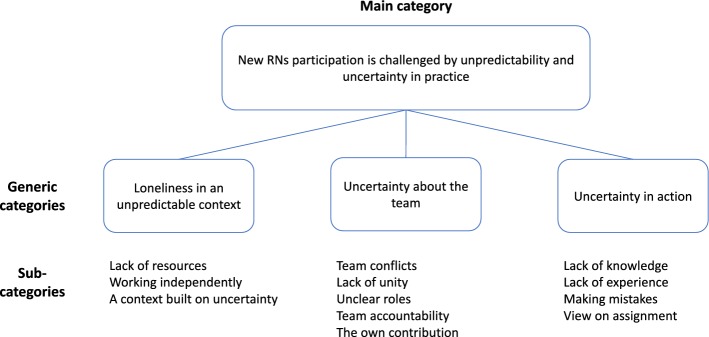
Illustration of the findings

The findings are presented as a main category and three generic categories (Fig. 2). To strengthen credibility, quotations are used to illustrate the findings.

## Findings

It was found that the social learning theory of CoP could be used to explore challenging encounters experienced by RNs during their first year in the EMS to a great extent. Statements that were not related to the challenge of a CoP were sorted as “other challenges”. All other challenges were about the EMS context.

### New RNs’ participation is challenged by unpredictability and uncertainty in practice

The main category describes that the new RNs’ participation is challenged by unpredictability and uncertainty in practice and constitutes actions and relationships that simultaneously occurred in the three dimensions of the CoP. The RNs did not know what to expected of themselves nor other health care professionals; neither did they know when to except it. In this unpredictable practice, the RNs described being affected by uncertainty about whether or not they could trust their own competence or that of others. Feeling uncertain and having only the EMT colleague to ask for support was reflected in uncertainty in action, i.e. how to handle situations.

### Loneliness in an unpredictable context

The EMS was described as a context where unpredictable situations occurred. When the unexpected happened, the RNs described the challenge of having to make independent decisions with limited support and resources. Adding to the uncertainty was receiving inadequate or incomplete information from the dispatch centre.

*We got a call…level four I think it was, a child that was crying, that’s all information we got, so we go there and…find an unconscious, pale, child laying on the floor…we panicked, grabbed the child and ran down to the car* (Informant 30).

Data shows a great diversity of encounters that EMS professionals need to be prepared for during their first year. These encounters ranged from making a decision to slow down and just hold an old man’s hand while he was taking his last breaths, to being directed to a night-time gun-shooting. Some RNs described situations that they knew they might encounter but had not yet encountered. The main reason for this uncertainty being described as challenging was that the RNs had no idea of how they would react, for example when encountering a drowning child or someone who had jumped in front of a train.

In the EMS, the RNs described not knowing whether or not there were more available resources when they arrived as the first ambulance at an accident or when attending a critically ill patient miles away from nearest hospital.

*It was rough, […] being the person that had to make the decision…* (Informant 12).

Being the only RN, they reflected that they had to be able to work independently even though they did not feel competent enough to do so. This included having to make autonomous medical or logistic decisions or deciding which patient to attend to first in a traffic accident.

### Uncertainty about the team

In the unpredictable context, the mutual engagement in patient care was affected by uncertainty in team roles. Not knowing what to expect of the colleague or what was expected of oneself led to conflicts within the team and a sense of non-existent team unity.

*I didn´t have a colleague, I just had a person that drove around with me in the car* (Informant 10).

The RNs were placed with different colleagues almost every day. When there were different levels of formal education and levels of experience, or when the colleague was new to the EMS the roles in the team were unclear and conflicts occurred. The RNs also reported being yelled at by a physician for not having done what the physician ordered them to do, or colleagues in assisting ambulances condescendingly commenting on decisions the RN had made. Colleagues not willing to support a new RN showed this by a neglectful attitude or by departing from guidelines without giving an explanation as to why.

*Eh it´s a mentality like, we´ll do it this way…and if I say But the guidelines say…they´ll be like…Yeah well now we do it this way* (Informant 10).

In another situation one RN even described having to take on the role of being responsible for another newly employed RN´s orientation.

*I mean, I had no idea either about what she knew or how I was supposed to teach her about how it is done in the ambulance* (Informant 16).

### Uncertainty in action

Conflicts could also arise in terms of negotiation about the joint enterprise, when new RNs and the more experienced colleague did not share the same view of treatment, ethical values or assignments.

*We got a level 1 call and it was a 30*-*year*-*old man with sudden, severe headache…my reaction was that this could be something serious…and my colleague said, this is probably psychological* (Informant 10).

Sometimes the colleague did not show respect or trust the RN’s competence and since they were uncertain about their own competence, the RN would “give in” to the colleague. When this led to wrong assessments or treatments, the RNs blamed themselves for not being strong enough to stand up for their own beliefs.

*We discussed it but he didn´t want to change his mind, so eventually I gave in and this still irritates me […] I felt so bad coming in to the hospital with this patient not immobilised, it was so embarrassing* (Informant 4).

The conflict of time taking precedence over necessary assessments and treatments was an experience that was described as well.

*On this one particular day my colleague was going to (an event) after the shift and was very stressed about this…I noticed because directly after reading the assignment he said, We´ll go to (local hospital) with this right? But I felt that this looked like a major trauma and our guidelines then say we should go to (region hospital)* (Informant 11).

Many of the situations regarded first-time encounters, such as the first cardiac arrest, the first time using some of the medical equipment or the first time giving a specific medication. When faced with first-time encounters, a lack confidence in themselves, or their colleagues, coupled with insufficient support from guidelines, the RNs found themselves in uncertain and uncharted territory.

*I don’t know, I did not know what to do…I had no idea…those 30* *minutes were the worst in my entire life…because I was so new…* (Informant 21).

When not knowing what to do and without the colleague or guidelines to provide assistance, there was no shared repertoire and the RNs had to create a repertoire themselves. Sometimes this led to the experience of sub-optimal treatments or assessments being made.

In terms of self-perceived lack of experience and knowledge, believing to have done something wrong or having made an erroneous assessment was described with self-criticism and remorse.

*I shouldn´t have done that; if I had gotten her to the hospital faster then she might have survived…* (Informant 5).

## Discussion

The findings display a variety of different encounters that were considered as challenging by the new RNs, challenges that could relate to the theory of CoP and challenges about the EMS context. The challenges evolved around the unpredictable context and uncertainty within the team and in action. The challenge of the unpredictability that frames an EMS context has been highlighted in prior research (Sundström and Dahlberg 2012). Unpredictability has also been identified as having negative influence on new professionals’ transition (Sutton and Griffin 2004; van der Werff and Buckley 2017). According to the theory of CoP, a person’s professional development can never be isolated from the world in which he/she acts (Lave 2009). However, the different prerequisites of a context and how these prerequisites affect professional development is not in focus in the theory of CoP. In the light of our findings, one way of preparing new professionals both for the EMS and other professional practices is by highlighting the particular prerequisites of the new professional context. Preparing new professionals for an unpredictable context may be challenging but nonetheless important.

The informants also described uncertainty concerning what to expect from their colleagues. Since professionals in the EMS in this study often had different levels of formal education than the other member of their team, the roles were unclear and this led to conflicts. In light of CoP one could view this result in two ways. Firstly, even though the mutual engagement revolved around patient care there were divergent views concerning the course of the assignment. This could indicate that in the EMS team RNs and EMTs belong to two different CoPs parallel to the common one and therefore collide.

In hospital settings, teams are larger, resources are greater and there is often someone else to turn to if a conflict arises (Mausz and Tavares 2017). According to Wenger (2000) people must know each other well enough to interact productively and know whom to call for help or advice. This might be difficult when being new to a community. Working in teams of two, as in the EMS also limits the options of whom to ask for help or advice and could be an explanation as to why conflicts arose in the EMS team.

Secondly, another way of viewing conflicts is as a natural process of transition and a part of being new to a CoP (Andersson and Edberg 2010; Barnes 2015; Chang and Hancock 2003; Rush et al. 2014). According to Lave and Wenger (1991), conflicts are a natural part of development and are generated when new professionals move from legitimate peripheral participation towards full participation. A conflict in itself should therefor neither be seen as positive nor negative. It is the meaning derived from the conflicts that will influence whether development will be creative or lead to inbred failure (Harenčárová 2017; Rush et al. 2014; Wenger 1998). Some conflicts described by the informants in this study were based on bullying behaviour by the experienced colleagues. The meaning derived from conflicts based on distrust, neglect or when a new professional is left unsupported is most often negative and does not lead to creative professional development (Pfaff et al. 2014; Rush et al. 2014). There might also be a risk when experienced colleagues show lack of respect for the new RN’s competence possibly resulting in new RNs refraining from seeking support in the future. Whichever way one views the challenges of conflicts, the findings presented here underscore the vulnerability of having only one colleague. It also stresses the importance of that colleague being trustworthy, respectful and able to support the new RN. Future research that focus on culture and attitudes of experienced professionals towards new colleagues in the EMS is needed to increase the knowledge about being new to the EMS context.

Another challenge of conflicts is that team collaboration may be affected. From the viewpoint of a CoP, new members in a community bring new ways of looking at the world that might illuminate limitations or possibilities that were previously unknown to the community (Wenger 2000). These new influences can either be accepted and lead to the community being redefined, or it can be suppressed. For a community to be redefined and for its members to develop, people need to be able to reflect on what they do, how and why (Schön 1983; Wenger 2000). Reflectivity, both on an individual level and within the team has been discussed as prerequisites for successful team collaboration (Conte et al. 2015a). Team collaboration is an important part of professionals’ engagement in practice and improves health outcomes (Conte et al. 2015a, b; World Health Organization 2010). The benefits of formal peer-support, for example mentorship, have been acknowledged in prior studies to support a new professional’s development and strengthen the sense of team unity (Chen and Lou 2014; Kalen et al. 2012; Rush et al. 2014). Even though Andersson and Edberg (2010) suggest that development occurs regardless of formal support, our study’s findings indicate that mentorship initiatives may be beneficial in contexts like the EMS.

The findings also showed that new professionals do not trust their own competence and when mistakes were made this was described with self-criticism and remorse. This part of the findings is in line with the findings of Wihlborg et al. (2016) that conclude that the way EMS professionals perceive their own competence is closely linked to patient outcome. Self-confidence influences how situations are conceived and an individual’s self-confidence can be affected by feedback and by gaining experience (Ortiz 2016). The need for feedback has been stressed before, and this study further highlights the importance of enabling EMS professionals to get feedback on their actions and development (Hörberg et al. 2017a, b; Smith et al. 2013).

### Strengths and limitations

The trustworthiness of a qualitative content analysis study is established by scrutinising credibility, dependability, conformability, transferability and authenticity in all three phases of the analysis process, i.e. in the preparation, organising and reporting phases (Elo et al. 2014; Lincoln and Guba 1985). Following the framework presented by Elo and Kyngäs (2008) provided a systematic and organised analysis process. Individual, telephone interviews were chosen since talking about challenging encounters may be sensitive to talk about in a group and may affect the individual’s answer. All three phases of the analysis were reported in a descriptive manner using illustrations and quotes to enabling the reader to determine dependability and transferability of the data. During the organisation phase, there is always a level of interpretation. However, the authors met regularly to discuss the analysis process and the mix of authors’ scientific knowledge and professional background were considered in order to reduce the risk of preunderstanding affecting the analysis. This study is also strengthened by the wide range of informants, representing both rural and urban areas. Even though the study took place in Sweden, international literature that relates to the study aim and the theoretical framework of CoP was used to increase the transferability of the findings. Authenticity refers to the extent to which authors consider a range of realities. Different realities were considered in the analysis process and presented in the discussion of the findings.

Using the theory of CoP is one way of conceptualising professional development (Wenger 1998). By using CoP, we do not mean to say that other theories of learning are less valid.

This study has been reported according to the Consolidated Criteria for Reporting Qualitative Studies (COREQ) checklist (Tong et al. 2007).

## Conclusion

By exploring challenging encounters using the theory of communities of practice, this study concludes that the challenges new RNs in the EMS encounter stems from challenges in mutual engagement, joint enterprise, a shared repertoire and from contextual prerequisites. The solitary working environment of the EMS is in itself a challenge that is exacerbated by the unpredictable nature of environments and situations. Adding to the personal challenges of transition were challenges such as conflicts or bullying behaviour from that one colleague or other experienced colleagues.

The findings of this study can be used when designing formal support models for new professionals in practices like the EMS and other ambulatory care practices. Mutual engagement and a joint enterprise can for example be supported when the new RNs feel trusted and respected by their colleagues and when the roles and view on assignment are clear within the team. Clear role descriptions seem to be of particular value when the level of formal education differs between the team members. Shared repertoire, i.e. ways to handle a new encounter, can be supported through formal feedback and having a trusted colleague.

New RNs’ professional development can also be supported if the RNs are being prepared for the specifics of the context they are new to. Having realistic expectations on the new professional practice may reduce the sense of uncertainty that arises when unpredictable events occur. Further research on how formal support models based on the understanding of socio-cultural learning can support professional development in organisations like the EMS is needed.
